# Adenoviral E3/49K: CD45-targeted immune evasion and therapeutic potential

**DOI:** 10.3389/fcimb.2026.1878561

**Published:** 2026-07-22

**Authors:** Mark Windheim, Khadija Aichane-Simon

**Affiliations:** Institute of Cell Biochemistry, Hannover Medical School, Hannover, Germany

**Keywords:** Ad19a, adenovirus, CD45, E3/49K, HAdV-D64, immune evasion, sec49K, tyrosine phosphatase

## Abstract

Human adenoviruses (HAdVs) cause a broad spectrum of diseases, ranging from mild respiratory and gastrointestinal infections to severe, sometimes even fatal illness in immunocompromised individuals. Species D adenoviruses represent the largest group, possibly driven by strong evolutionary pressure, particularly within the highly variable early transcription unit 3 (E3), which encodes immunomodulatory proteins. Among these, E3/49K is unique to species D and utilizes a particularly intriguing immunomodulatory strategy. It is a heavily glycosylated transmembrane protein that undergoes proteolytic shedding to release the soluble ectodomain (sec49K), the only known secreted HAdV protein. Both the membrane-anchored and soluble forms target the leukocyte-specific receptor-like protein tyrosine phosphatase CD45, a key regulator of immune cell activation. E3/49K binds CD45 with high affinity via two domains, inducing dimerization and inhibiting its phosphatase activity, thereby suppressing activation of T, B, and NK cells. The species specificity and potent immunomodulatory function of E3/49K underscore its role in adenoviral adaptation and highlight its potential for therapeutic immunosuppression and cancer therapy.

## Introduction

Human adenoviruses (HAdVs) are associated with a wide range of diseases, from respiratory and ocular infections to gastroenteritis. Usually these infections are self-limiting in immunocompetent individuals, but they can become severe or even be fatal in immunocompromised patients (Berk, 2013; Wold and Ison, 2013; MacNeil et al., 2023). More than 100 HAdV types have been identified and are classified into species A through G. The great majority (>70 types) belong to species D (Ismail et al., 2018), in which homologous recombination is a major driver of evolution (Robinson et al., 2011, 2013). Because species A and D lack simian relatives, they may have originated within the human population (Hoppe et al., 2015). Presumably, the large number of types arose through host selection pressures promoting the emergence of new lineages. Despite their diversity, only a few HAdV-D types cause epidemic keratoconjunctivitis (EKC), a highly contagious and severe ocular disease. Since all species D viruses use CD46 and sialic acid as primary entry receptors, receptor usage alone cannot explain the EKC-causing phenotype (Arnberg, 2012). Instead, the early transcription unit 3 (E3) has been implicated in viral pathogenicity *in vivo*, and may therefore contribute to virus specific-diseases such as EKC (Windheim et al., 2004).

## Early transcription unit 3: the immunomodulatory arsenal of adenoviruses

HAdVs have evolved various strategies to evade the host immune system, many of which are encoded within the E3 region. Although this region is dispensable for viral replication *in vitro* (Berkner and Sharp, 1983), it plays a critical role for viral pathogenicity *in vivo* (Ginsberg et al., 1989), highlighting its importance during infection. Functional studies have shown that E3 proteins suppress host immune responses and interfere with the recognition of infected cells (Burgert et al., 2002; Windheim et al., 2004; Oliveira and Bouvier, 2019).

The E3 region is highly divergent and different species vary markedly in both the size and the composition of its encoded proteins (Burgert and Blusch, 2000; Burgert et al., 2002; Windheim et al., 2004). Notably, species D has the largest and most complex E3 region, encoding eight open reading frames (ORFs), which may play an important role for its adaptation to human hosts (Hoppe et al., 2015). Moreover, the E3 region exhibits the highest nucleotide diversity among HAdVs (Robinson et al., 2011, 2013; Ismail et al., 2018, 2019). This aligns well with its role in immune evasion and underscores that the acquisition of immunomodulatory functions and their adaptation to the host under immune pressure are a major driving force of adenovirus evolution.

The discovery that the E3/19K protein blocks the presentation of viral antigens by major histocompatibility complex class I (MHC class I) molecules through binding and retention in the endoplasmic reticulum (ER) was the first demonstration of an immunomodulatory activity encoded by the E3 region (Burgert and Kvist, 1985; Paabo et al., 1986; Burgert and Kvist, 1987). In addition, E3/19K down-regulates the NKG2D ligands MHC class I-related chain A (MICA) and MICB (McSharry et al., 2008; Sester et al., 2010). Thus, E3/19K subverts both T-cell recognition and natural killer (NK)-cell activation. Subsequently, additional E3-encoded immunomodulatory proteins have been identified, namely 49K, 10.4K (RIDα), 14.5K (RIDβ), 6.7K, and 14.7K.

The RIDα/RIDβ proteins (also called E3/10.4K/14.5K) inhibit death receptor-mediated apoptosis by targeting Fas (CD95), tumor necrosis factor (TNF)-related apoptosis-inducing ligand (TRAIL)-receptor 1 (TRAIL-R1) and TRAIL-R2 (in concert with E3/6.7K) for lysosomal degradation (Shisler et al., 1997; Elsing and Burgert, 1998; Tollefson et al., 1998; Benedict et al., 2001; Tollefson et al., 2001). The RIDα/RIDβ complex also downregulates the epidermal growth factor receptor (EGFR) from the cell surface, possibly to suppress pro-inflammatory host responses (Carlin et al., 1989; Tollefson et al., 1991). The 14.7K protein inhibits TNF-mediated apoptosis by blocking TNFR1 internalization and downstream death signaling, thereby promoting immune evasion (Gooding et al., 1988, 1990; Horton et al., 1991; Tufariello et al., 1994; Schneider-Brachert et al., 2006). We studied the E3/49K protein (also called CR1-β) and uncovered a novel mechanism of adenoviral immune evasion (Deryckere and Burgert, 1996; Blusch et al., 2002; Windheim and Burgert, 2002; Windheim et al., 2013, 2016, 2023).

## E3/49K: a membrane-anchored protein and its secreted ectodomain sec49K

**Figure 1:**
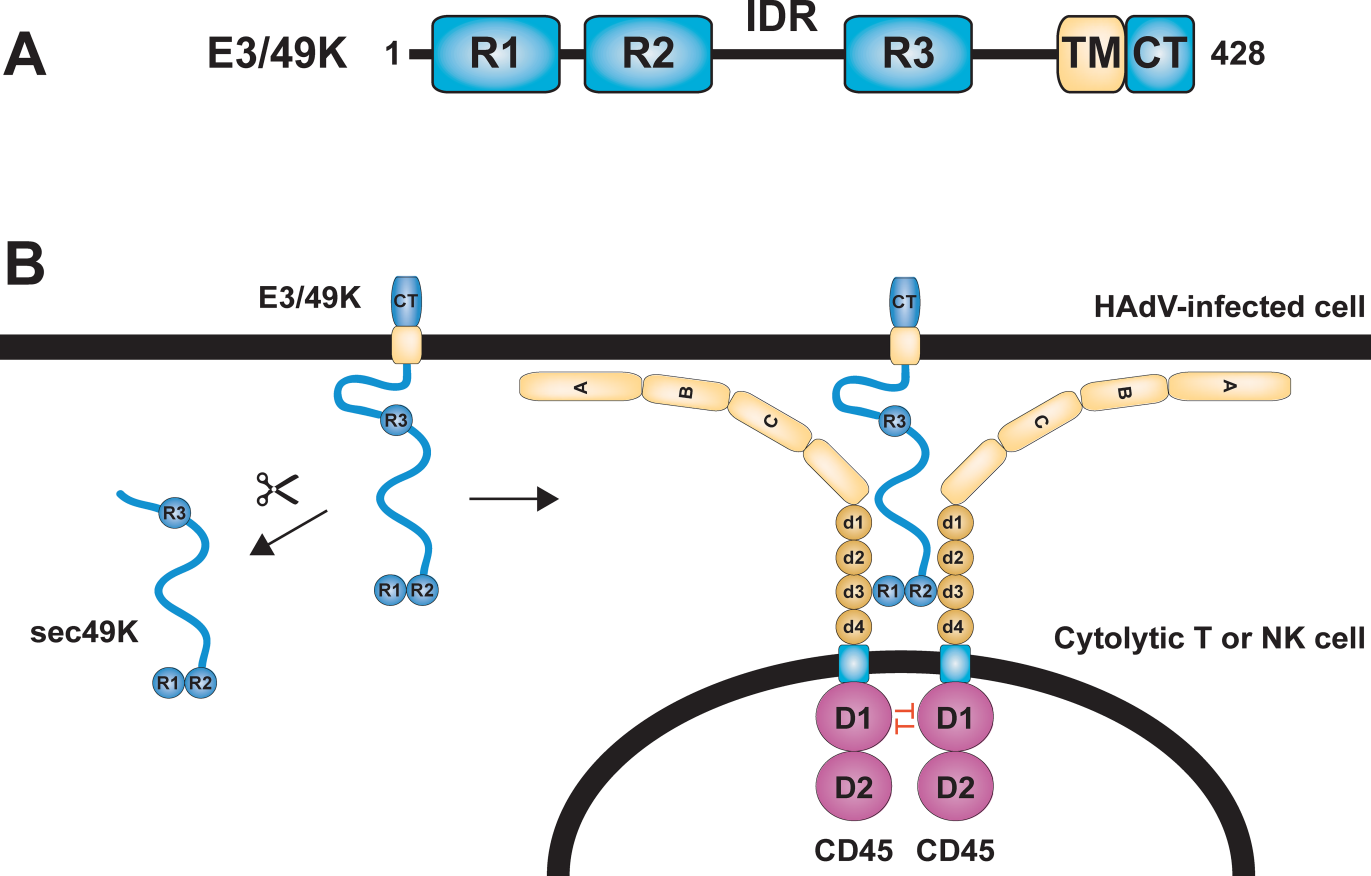
Mechanism of E3/49K-mediated CD45 inhibition. **(A)** Full-length transmembrane E3/49K consists of a short cytoplasmic region (CT) containing motifs for endocytosis and intracellular sorting, a transmembrane domain (TM), and a large luminal N-terminal region comprising three CR1 domains with an immunoglobulin-like fold (R1–R3). R2 and R3 are separated by a ~70−residue intrinsically disordered region (IDR). **(B)** At the cell surface, metalloproteases release the soluble ectodomain sec49K through shedding. Both sec49K and the full-length E3/49K bind the d3 domain of the receptor-like tyrosine phosphatase CD45, inducing dimerization and inhibition of its intracellular phosphatase activity. The IDR in E3/49K presumably functions like a flexible linker or “fishing line”, bridging the distance to the CD45 binding site on cytolytic immune cells (adapted from Windheim et al., 2023).

E3/49K was first identified in the E3 region of EKC-causing HAdV-D64 (Deryckere and Burgert, 1996) and is specific to species D adenoviruses (Burgert and Blusch, 2000; Burgert et al., 2002). Therefore, it may have contributed to the emergence of species D adenoviruses within the ancestral human reservoir (Hoppe et al., 2015). E3/49K is a highly glycosylated type I transmembrane protein with a short cytoplasmic tail and a large mature molecular mass of 80–100 kDa (**Figure 1A**). It harbors 14 potential N- and three O-glycosylation sites, of which approximately 12–13 N-linked sites are occupied (Windheim and Burgert, 2002). The extracellular domain (ECD) is composed of three homologous domains, referred to as conserved regions 1-3 (R1-3). These regions show homology to domains found in other HAdV E3 proteins and the human cytomegalovirus (HCMV) RL11 gene family, and were designated CR1 for both virus families (Davison et al., 2003). The three CR1 domains of E3/49K adopt an immunoglobulin-like fold, suggesting that the observed homology is based on structural similarities (Windheim et al., 2023; Borowska et al., 2024). Notably, the R1 and R2 domains are divided by only 16 residues, whereas R2 and R3 are separated by a long intrinsically disordered region (IDR) of approximately 70 residues (**Figure 1A**).

At steady state, E3/49K localizes to the endoplasmic reticulum, Golgi/trans-Golgi network, early endosomes, and the cell surface, reflecting its dynamic intracellular trafficking (Windheim and Burgert, 2002; Windheim et al., 2016). The LL and the YXXΦ (Φ, hydrophobic amino acid) motifs within the 19-residue cytoplasmic tail serve as the primary internalization signals mediating endosomal/lysosomal sorting and trafficking via clathrin adaptor protein complex-1 (AP-1) and AP-2 interaction (Windheim et al., 2016).

Interestingly, E3/49K is cleaved by a matrix metalloprotease at the cell surface, releasing the small membrane-anchored C-terminal fragments and the large secreted ectodomain sec49K (Windheim et al., 2013). A disintegrin and metalloprotease 10 (ADAM10) and ADAM17 have been identified as the principal sheddases (Windheim et al., 2016). Notably, ectodomain shedding even occurs in porcine cells (Pokoyski et al., 2023). Sec49K is the only HAdV protein known to be secreted to date. Therefore, E3/49K exemplifies a novel immune evasion strategy by targeting uninfected immune cells rather than acting exclusively within infected cells. This is mediated through binding to the leukocyte-specific receptor-like protein tyrosine phosphatase CD45 (Windheim et al., 2013, 2023).

## CD45: the molecular target of E3/49K

CD45 (Protein Tyrosine Phosphatase Receptor Type C, PTPRC) is highly expressed on all leukocytes, accounting for up to 10% of the total surface protein (Thomas, 1989). The CD45 gene consists of 35 exons. Alternative splicing of exons 4, 5, and 6 generates multiple CD45 isoforms that differ in the ECD by the presence or absence of regions A, B, and C. Distinct types of immune cells can be distinguished based on their expression of specific CD45 isoforms (Trowbridge and Thomas, 1994; Hermiston et al., 2003; Rheinlander et al., 2018; Al Barashdi et al., 2021). In contrast, the membrane-proximal extracellular domains d1 to d4 and the intracellular tandem protein tyrosine phosphatase domains, D1 and D2, are present in all isoforms (Chang et al., 2016). Of these, only the phosphatase domain D1 is catalytically active (Streuli et al., 1990; Nam et al., 2005).

The extracellular domains of CD45 are highly glycosylated: N-glycans are primarily found in the membrane-proximal domains d1–d4, while O-glycans are mainly located in the membrane-distal A, B, and C regions (Earl and Baum, 2008). Various lectins, such as galectins, CD22, mannose receptor, or the placental protein 14 (PP14) can bind CD45 (van der Merwe et al., 1996; Martinez-Pomares et al., 1999; Symons et al., 2000; Ish-Shalom et al., 2006). But to date, no high-affinity physiological ligand for CD45 has been identified. Instead, CD45 may function in a ligand-independent manner in T cell receptor (TCR) signaling in line with the kinetic segregation model, which is based on size-dependent exclusion from TCR-pMHC close contacts (Davis and van der Merwe, 2006). The rigid extracellular region of CD45 with an effective height of ~20–30 nm, depending on the isoform, extends beyond the TCR-pMHC complex and thereby excludes CD45 from the contact site (Chang et al., 2016).

CD45 function has been studied extensively in T cells, where it is critical for activation and proliferation (Pingel and Thomas, 1989; Koretzky et al., 1990). It plays a central role in signaling pathways initiated by the TCR (Courtney et al., 2018; Gaud et al., 2018). Specifically, CD45 triggers TCR signaling by the dephosphorylation of the inhibitory tyrosine residue (pY505) near the C-terminus of the Src family lymphocyte-specific kinase Lck (Sieh et al., 1993), enabling subsequent phosphorylation of the activating tyrosine in the activation loop (Y394) for maximal Lck activity (Philipsen et al., 2017). Strikingly, the activating pY394 is also a substrate of CD45 phosphatase activity (D'Oro and Ashwell, 1999), supporting the idea that CD45 functions as a rheostat that fine-tunes Lck activity and TCR signaling (McNeill et al., 2007) as well as a “gatekeeper” by maintaining a pool of active Lck and suppressing weak signals (Courtney et al., 2019). However, CD45 is not only important for T cell activation but also plays a critical role in other leukocytes (Rheinlander et al., 2018). Notably, CD45 expression has been reported to be dysregulated in leukemia and lymphoma (Saint-Paul et al., 2016; Perron and Saragovi, 2018; Al Barashdi et al., 2021).

## How E3/49K inhibits CD45

The mechanism by which E3/49K binding modulates CD45 function remained unclear until recently. We have now shown that both the R1 and the R2 domain of E3/49K bind the d3 domain of CD45, thereby enforcing its dimerization (Windheim et al., 2023). Because the d3 domain is present in all CD45 isoforms, both E3/49K and its secreted form, sec49K, interact with all isoforms (Windheim et al., 2023; Borowska et al., 2024), with binding affinities in the subnanomolar range (~0.1 to 0.3 nM) (Martinez-Martin et al., 2016; Borowska et al., 2024).

This model has been corroborated by cryo-electron microscopy (cryo-EM) analysis of the E3/49K–CD45 complex (Borowska et al., 2024). In the cryo-EM structure, the R1 and R2 domains enable a single sec49K molecule to bridge two CD45 proteins, inducing the formation of an asymmetric dimer. The R3 domain of sec49K contacts the same d3 domain of CD45 as the R2 domain, but from the opposite site. However, in surface plasmon resonance analysis no binding of the R3 domain to the CD45 ECD was detected, suggesting that the R3–d3 contact observed in the cryo−EM structure may be of low affinity or artefactual.

Although the R3 domain is dispensable for CD45 inhibition *in vitro* (Windheim et al., 2023; Borowska et al., 2024), it may stabilize the sec49K–CD45 complex or influence the accessibility of the metalloprotease cleavage site. While the existence of an unidentified cell surface binding partner cannot be formally excluded, the absence of detectable sec49K binding to CD45-deficient Jurkat T cells and Ramos B cells, as well as to non-hematopoietic cells (Windheim et al., 2013; Hildenbrand et al., 2024), argues against the presence of a cell surface receptor other than CD45. In contrast, extracellular matrix components and associated lectins remain potential interaction partners for the R3 domain.

Sec49K-mediated dimerization of CD45 likely positions the intracellular phosphatase domains into close proximity, thereby sterically hindering substrate access. This dimerization-induced inhibition of CD45 phosphatase activity has been demonstrated to impair TCR signaling by preventing Lck dephosphorylation at pY505 and subsequent phosphorylation at Y394, as well as ZAP70 phosphorylation, and by reducing downstream phosphorylation of extracellular signal-regulated kinases 1 and 2 (ERK1/2), suggesting disrupted LAT (linker for the activation of T cells) signalosome assembly (Windheim et al., 2013, 2023; Borowska et al., 2024). In B cells, sec49K has been shown to impair the B cell receptor (BCR)-dependent phosphorylation of the spleen tyrosine kinase (Syk) and ERK1/2 (Hildenbrand et al., 2024), and is likely to modulate additional substrates across other leukocyte subsets. Ultimately, these effects suppress activation of T, B and NK cells, impairing key functions such as cell proliferation, cytotoxicity, antibody production and pro-inflammatory cytokine secretion (Windheim et al., 2013; Martinez-Martin et al., 2016; Pokoyski et al., 2023; Borowska et al., 2024; Hildenbrand et al., 2024).

Negative regulation of receptor protein tyrosine phosphatases by dimerization is a well-established concept (Weiss and Schlessinger, 1998; Welsh et al., 2021). Consistent with this model, dimerization-induced inhibition of CD45 phosphatase activity has been proposed previously (Takeda et al., 1992; Desai et al., 1993; Majeti et al., 1998). However, in the absence of a known physiological ligand, the mechanism regulating CD45 dimerization remains enigmatic. Nevertheless, E3/49K appears to hijack this dimerization mechanism to inhibit CD45 phosphatase activity.

The N-terminal R1 and R2 domains of E3/49K mediate CD45 binding and are preceded by an extended IDR of 60–70 residues located between the R2 and R3 domains. In the membrane-anchored form, this architecture of E3/49K resembles a “molecular fishing rod”, in which the IDR acts as a “fishing line” that bridges the intercellular gap between the infected cell and an adjacent immune cell, thereby positioning the R1 and R2 “baits” to capture CD45 on the immune cell (Windheim et al., 2023). This design suggests that not only the secreted form, sec49K, where the IDR does not contribute to CD45 binding, but also the membrane-bound E3/49K is functionally important. This may explain why HAdVs have evolved a transmembrane protein that is shed rather than producing a solely secreted protein.

Furthermore, distinct, albeit possibly overlapping, functions of the membrane bound and soluble E3/49K can be envisaged. The membrane bound E3/49K may preferentially target immune cells that directly attack the infected cell, such as cytotoxic T lymphocytes or NK cells. The soluble sec49K could, in addition, inhibit CD45 positive cells at a greater distance, for example T helper cells or B cells.

Notably, E3/49K is the most polymorphic viral protein among species D adenoviruses, suggesting that it is under strong evolutionary selection pressure imposed by host immune defenses (Robinson et al., 2013). Strikingly, also the ECD of CD45 is hypervariable and has undergone strong positive selection in humans (Borda et al., 2020), primates (Filip and Mundy, 2004) and cattle (Ballingall et al., 2001), likely driven by pathogens. Together, these observations suggest that E3/49K and CD45 are key participants in an ongoing virus-host arms race.

The E3/49K ECD is most similar to the species B 20.1K (CR1-β) and 20.5K (CR1-γ) proteins (Deryckere and Burgert, 1996), which interact with each other (Lakshmi Narayan and Kajon, 2020) but do not bind CD45 (Martinez-Martin et al., 2016). Notably, no HAdV species other than species D encodes a CD45-binding protein (Hildenbrand et al., 2024). A few other viruses have been reported to target CD45, yet appear to use different strategies. The Roseoloviruses downregulate CD45 through transcriptional suppression (Whyte et al., 2021). Mouse cytomegalovirus (MCMV) m42 induces the lysosomal degradation of CD45 (Thiel et al., 2016) and the HCMV UL11 protein as a recombinant Fc-fusion protein increased interleukin-10 (IL-10) secretion (Gabaev et al., 2011, 2014; Zischke et al., 2017; Osanyinlusi et al., 2022). However, Fc-mediated homodimerization may induce artifactual pUL11 dimers that drive CD45 dimerization and inhibition. Thus, whether monomeric pUL11 acts in trans or, alternatively, in cis on infected cells remains unclear.

## From virus to cure: therapeutic potential of E3/49K

Through millions of years of coevolution with their hosts, viruses have evolved diverse strategies to evade and modulate immune responses, thereby optimizing their replication and persistence. Adenoviruses are a well-studied example, encoding a broad repertoire of immunomodulatory proteins that target innate and adaptive immune pathways. As products of long-term host-pathogen coevolution, these viral mechanisms provide valuable insight for the rational design of novel therapeutic strategies.

**Figure 2:**
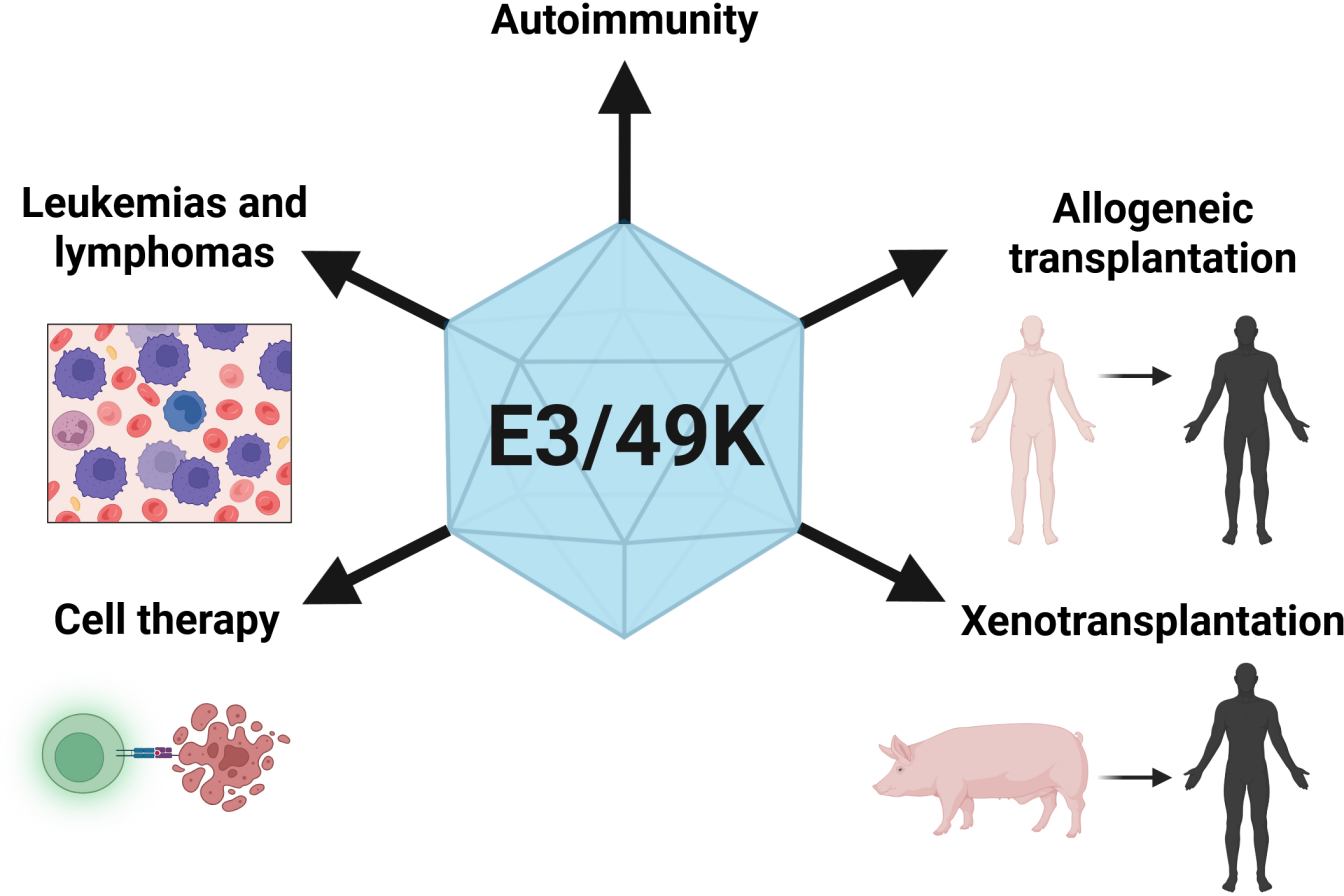
Therapeutic applications of E3/49K-mediated CD45 inhibition. Recombinant sec49K, full-length E3/49K, and cleavage−resistant variants offer a broad range of therapeutic opportunities. Potential applications include: (i) targeted immunosuppression for autoimmune and chronic inflammatory diseases; (ii) protection of therapeutic cells against immune rejection; (iii) graft protection in allogeneic and xenogeneic transplantation; and (iv) CD45−targeted therapy for leukemias and lymphomas with aberrant CD45 expression. Created in BioRender. Windheim, M. (2026) https://BioRender.com/2gnw2au.

Our investigations into the function of the E3/49K identify CD45 as an attractive therapeutic target. Because of its broad expression across leukocytes, CD45 is particularly well suited for immunotherapeutic strategies (Dahlke et al., 2004). Since low-molecular-weight phosphatase inhibitors often lack selectivity, biologics that bind CD45 extracellularly and inhibit its intracellular phosphatase activity represent a promising alternative (Senis and Barr, 2018). Consequently, E3/49K and its secreted form, sec49K, may provide novel strategies to suppress pathological immune activation in autoimmune and chronic inflammatory diseases, as well as in transplantation settings (**Figure 2**). Beyond its role in immune modulation, CD45 also represents a promising target for cancer therapy, as it has been reported to be aberrantly expressed in leukemia and lymphoma (Saint-Paul et al., 2016; Perron and Saragovi, 2018; Al Barashdi et al., 2021).

While the therapeutic potential of E3/49K is promising, long-term administration of a viral protein may elicit immune responses. To mitigate this limitation, alternative formats such as humanized variable domain of heavy-chain-only antibodies (VHHs, nanobodies), diabodies, or bispecific antibodies targeting CD45 may be employed. These engineered formats preserve the functional targeting of CD45 while reducing immunogenicity, thereby balancing translational potential with safety.

E3/49K-based strategies hold particular promise in transplantation. Soluble sec49K may be applicable in allogeneic settings, whereas expression of E3/49K in transgenic donor tissues, such as genetically modified pigs, could protect grafts in xenotransplantation. Notably, the interaction between E3/49K and CD45 is highly species-specific, with no detectable binding to porcine CD45, likely reflecting substantial interspecies variation in the ECD (Pokoyski et al., 2023). Human CD45 shares only ~40% sequence identity within the ECD with murine and porcine orthologs, whereas the intracellular region is highly conserved (~90% identity), suggesting strong evolutionary pressure on the ECD. Consequently, expressing E3/49K in transgenic pigs is unlikely to adversely affect the porcine immune system. Expression of E3/49K in porcine cells has been shown to reduce human anti-pig immune responses, supporting its potential use for xenograft protection (Pokoyski et al., 2023). This effect may be further enhanced through protein engineering approaches that eliminate proteolytic cleavage sites, thereby preventing shedding and promoting sustained surface expression.

Beyond transplantation, E3/49K may protect therapeutic cells from immune rejection. This includes applications in cell therapies based on embryonic stem cells, induced pluripotent stem cells, as well as chimeric antigen receptor (CAR) T-cell therapy and CAR NK-cell therapy (Bashor et al., 2022). Such approaches may enable the generation of “off-the-shelf” cell therapies.

Finally, CD45-targeting approaches may be exploited for active signaling modulation. Given that aberrant tyrosine phosphorylation underlies many inflammatory and oncogenic processes, CD45-binding domains derived from E3/49K or engineered mimetics could be used to enforce CD45 recruitment to receptor tyrosine kinases or other receptors, e.g., inhibitory NK receptors (Ren et al., 2022) and PD-1 (Fernandes et al., 2020). This strategy may enable targeted dephosphorylation of signaling molecules, thereby suppressing pathological signaling in immune cells as well as in leukemia and lymphoma.

## Concluding remarks

The study of viruses not only reveals how remarkably simple protein-coated RNA or DNA genomes can manipulate far more complex cells and organisms, but also uncovers evolutionary strategies that offer valuable insights for therapeutic innovation. The adenoviral E3/49K protein is a compelling example of this principle. Both the membrane−anchored form and the shed ectodomain, sec49K, protect infected cells by forming an “immune−shield”. This mechanistic insight highlights multiple therapeutic opportunities. Recombinant sec49K or engineered, cleavage−resistant E3/49K variants could selectively target CD45, potentially offering a highly specific alternative to low−affinity small−molecule phosphatase inhibitors. Future work may enable translation of this virus−engineered immune−modulator into next−generation immunotherapies.
